# Evaluation of Proton Therapy Accuracy Using a PMMA Phantom and PET Prediction Module

**DOI:** 10.3389/fonc.2018.00523

**Published:** 2018-11-13

**Authors:** Junyu Zhang, Yan Lu, Wenchien Hsi, Jiangang Zhang, Yinxiangzi Sheng, Leijun Shi, Weiwei Wang, Jiade Lu, Rong Zhou, Jingyi Cheng

**Affiliations:** ^1^College of Physical Science and Technology (College of Nuclear Science and Engineering), Sichuan University, Chengdu, China; ^2^Key Laboratory of Radiation Physics and Technology Ministry of Education, Chengdu, China; ^3^Department of Medical Physics, Shanghai Proton and Heavy Ion Center, Shanghai, China; ^4^Department of Radiation Oncology, Miami Cancer Institute Baptist Health South Florida, Miami, FL, United States; ^5^Department of Nuclear Medicine, Shanghai Proton and Heavy Ion Center, Shanghai, China; ^6^Department of Radiation Oncology, Shanghai Proton and Heavy Ion Center, Shanghai, China; ^7^Department of Nuclear Medicine, Shanghai Proton and Heavy Ion Center, Fudan University Cancer Hospital, Shanghai, China

**Keywords:** proton therapy, positron emission tomography (PET), PMMA phantom, depth verification, lateral verification

## Abstract

**Purpose:** Positron emission tomography (PET) scanning is a widely used method of proton therapy verification. In this study, a proton radiotherapy accuracy verification process was developed by comparing predicted and measured PET data to verify the correctness of PET prediction and was tested at the Shanghai Proton and Heavy Ion Center.

**Method:** Irradiation was performed on a polymethyl methacrylate (PMMA) phantom. There were two dose groups, to which 2 and 4 Gy doses were delivered, and each dose group had different designed dose depths ranging from 5 to 20 cm. The predicted PET results were obtained using a PET prediction calculation module. The measured data were collected with a PET/computed tomography device. The predicted and measured PET data were normalized to similar PET amplitude values before comparison and were compared using depth and lateral profiles for the position error. The error was evaluated at the position corresponding to 50% of the maximum on the PET curves. The mean and standard deviation were calculated based on the data sampled in the scoring area. Gamma index analysis is also applied in the comparison.

**Results:** In the depth comparison, the 2 and 4 Gy dose cases yielded similar mean depth errors between 1 and −1 mm, and the deviation was <2 mm. In the lateral comparison, the 2 Gy cases had a mean lateral error around 1 mm, and the 4 Gy cases had a mean lateral error <1 mm, with a standard deviation <1 mm for both the 2 and 4 Gy cases. All the cases have a gamma passing rate over 95%.

**Conclusion:** The comparison of these PMMA phantom cases revealed good agreement between the predicted and measured PET data, with depth and lateral position errors <2 mm in total, considering the uncertainty. The comparison results demonstrate that the PET predictions obtained in PMMA phantom tests for single proton beam therapy verification are reliable and that the research can be extended to verification in human body treatment with further investigation.

## Introduction

Proton therapy has already been confirmed to be an efficient method of solid tumor treatment (1). A proton beam can deposit most of its dose at the end of the beam range in the so-called Bragg peak and can reduce the dose deposited along the beam track in normal tissue. To achieve high accuracy, the Bragg peak must be within the tumor region, and some method is necessary to verify whether this requirement is met. A commonly used method is positron emission tomography (PET) (2–4), which can be employed to score the positron-emitting isotopes generated by the incident protons. The PET device collects positron annihilation signals from proton-activated isotopes such as C-11 and O-15, and the signal is then reconstructed to form an activity distribution image.

Although the generated positron-emitting isotopes can be scored, the nuclear reaction process is different from the proton energy deposition process. In addition, the depth dose range is different from the isotope generation range, due to the cross-section cut-off about 20–30 MeV (5) of the positron-emitting isotope-generating channel (6). A proton with this energy can typically still travel for a water equivalent depth of about 1 cm, this is why the activity depth cannot directly reflect the dose depth. One possible means of evaluating the dose delivery depth is to compare measured and predicted PET images. The predicted images can be generated by Monte Carlo simulation (2, 7). By simulating the treatment plan, both the dose and activity distributions can be scored. Monte Carlo simulation can provide all the desired information, and the accuracy of the results depends on the reference database and simulating primary number. However, it takes a long time to obtain reliable results via Monte Carlo simulation, and the simulated results strongly depend on the cross-section data (8). Frey et al. (9) provided a method of predicting PET activity distributions that takes much less time than Monte Carlo prediction, and a prediction script called PET-RV was developed based on this algorithm.

The objectives of this research were to verify the prediction accuracy in phantom cases with plan series and to develop a verification process. The results calculated using the PET-RV module were compared with the PET scanning data from the phantom irradiation experiment using a new statistical comparison method to assess the accuracy of the calculation results.

## Methods and materials

### Phantom irradiation and PET acquisition

**Figure 1:**
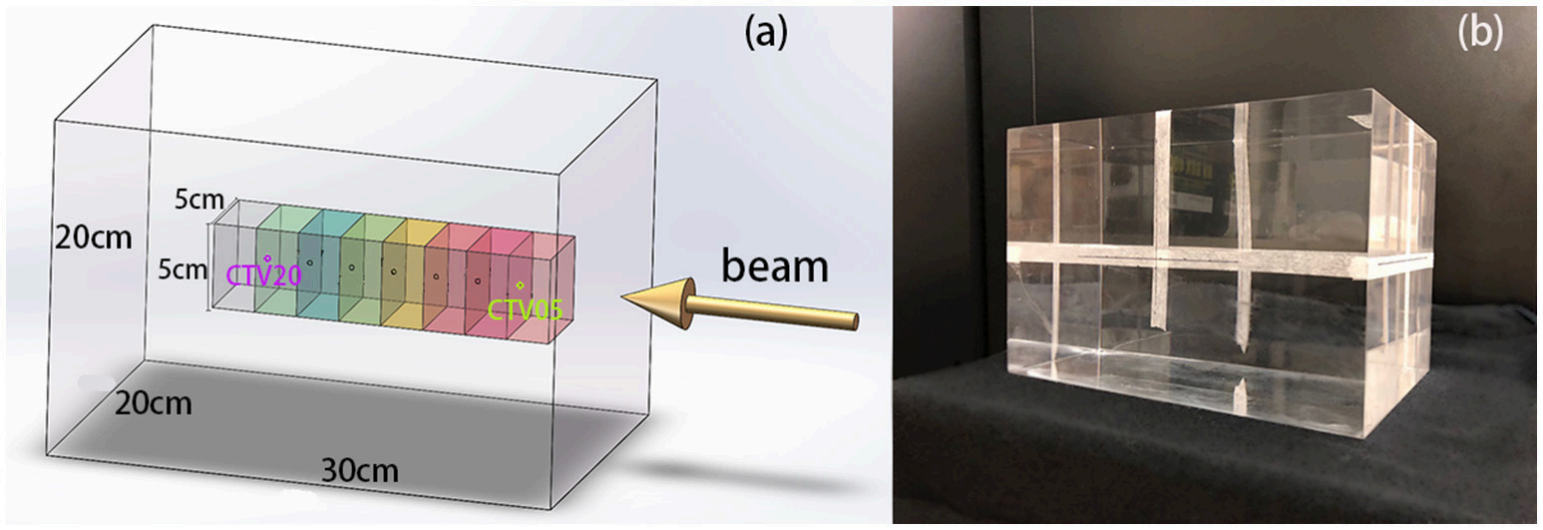
**(A)** 3D model of the phantom, CTVs, and beam direction. The center points of the CTVs are located along the beam line, with different distances to the beam entrance surface. **(B)** PMMA phantom used in the experiments.

In this experiment, we used polymethyl methacrylate (PMMA) as the material of the phantom. The elemental composition of PMMA is C_5_H_8_O_2_, its mass density is 1.19 g/cm^3^, and its stopping power relative to that of water is 1.156. This material is widely used in particle therapy experiments (10–12). The phantom was a transparent and homogeneous block with dimensions of 30 cm × 20 cm × 20 cm. In this cubic phantom, the clinical target volumes (CTVs) were designed as 5 cm × 5 cm × 5 cm cubes with their centers along the central beam line, and a depth series from 5 cm to 20 cm from the surface of the beam entrance in 2.5 cm steps, as shown in Figure 1.

We designed an irradiation plan series for all CTVs at different depths, with physical doses of 2 and 4 Gy, since a dose around 2 Gy is commonly used for clinical proton treatment in fractions. Following these experiments, we compared the PET-RV predictions to the experimental measurements and analyzed the differences between the distributions.

This experiment was conducted at Shanghai Proton and Heavy Ion Center, where a proton beam is generated by a linac-synchrotron combination similar to what is used at the Heidelberg Ion Therapy Center (13). The proton beam energy can be set between 48 and 221 MeV, corresponding to water equivalent depths from 2 to 31 cm. The dose depth in the PMMA phantom experiment was designed to be from 5 to 20 cm, a range that is entirely covered by the available beam energies. The proton particles were delivered spot by spot with pencil beam scanning (PBS), from the lowest energy layer to the highest energy layer. Depending on the dose that was required to be delivered in our experimental cases, the delivery time varied from 1 to 3 min.

The PET/computed tomography (CT) scanner was a Biograph mCT (14) produced by Siemens, which has four rings of 192 blocks in total, each of which contains 13 × 13 lutetium-oxyorthosilicate (LSO) crystals with dimensions of 4 mm × 4 mm × 20 mm. For this phantom experiment, the reconstructed PET images had 200 × 200 pixels per slice, the image pixel size was 4 mm × 4 mm, and the slice thickness was 5 mm. The pixels in the reconstructed CT images had dimensions of 0.98 mm × 0.98 mm, and again the slice thickness was 5 mm. Offline PET acquisition (4, 15) was used for data scoring. The cooling time from the end of irradiation until the beginning of acquisition was around 10 min. Typically, a cooling time between 10 and 15 min is suitable for most offline PET operation (12, 16), especially when PET acquisition is performed on a patient. The PET data acquisition took 30 min, and the collected data were reconstructed by conducting 2D ordered subset expectation maximization (OSEM) (17) in Na-22 mode to ensure that no decay time correction was applied. CT attenuation correction was also applied to make the reconstructed results fit the predicted results more closely.

### PET prediction using PET-RV

All of our data calculations, comparison, and analysis were performed using a RayStation Planning System (version 6.99). To predict PET results using PET-RV, a complete data set for treatment containing patient geometry information is necessary, which is often presented as CT data with structural information drawn by a doctor, as well, as an irradiation plan generated by a treatment planning system (TPS).

A proton treatment plan is the primary requirement for the script and contains information about the proton energy layers, spot distribution on each layer, and details of each spot. In addition, the spot scanning time course, including the temporal information about proton delivery, cooling, and acquisition duration, is necessary. Together with the activity decay and washout time parameters in different tissues, the temporal information is used to rescale the predicted PET values. Furthermore, all possible reaction channels during irradiation need to be considered in the calculations. Positron emitters such as C-11, N-13, O-15, P-30, and K-38 have different half-lives. In phantom cases, PMMA has three kinds of elements: C, O, and H, and the corresponding channels are (cross section with E>100 MeV): ^16^O(p,pn)^15^O (40 mb), ^16^O(p,αpn)^11^C (20 mb), ^12^C(p,pn)^11^C (40 mb), ^16^O(p,α)^13^N (10 mb). The cross section of these channels is reported in ICRU-report 63 (5). These channels should be enabled in the script.

The process through which PET-RV generates a predicted activity distribution is similar to that of a treatment planning system when finalizing a dose distribution based on a given plan. A look-up PET data table is constructed in place of a dose data table, and the corresponding data with the given particle energies, directions, and other parameters are drawn. This reconstruction process (9) with a look-up data table is much faster than Monte Carlo simulation and yields highly accurate results.

### Measured data smoothing and normalization

A co-registration between plan-CT and PET-CT is necessary before the comparison, since the PET prediction calculation is based on plan-CT and the measured PET data is acquired under the geometry frame of PET-CT. In our experiment, the CT co-registration is executed on the TPS platform, and the co-registration is processed based on HU value. This phantom with a regular geometry shape is set to a patient coordinate and correctly placed before CT scanning so the co-registration process will need only translation and the rotation is not required. For the patients' case, co-registration technology is well developed for clinical application, this will not be a problem in CT, as well as, the PET image.

The acquired PET data did not have a smooth distribution, Instead, there were numerous ripples in the curve due to the background noise and attenuation correction based on the CT Hounsfield unit value. Before the measured data could be compared with the predicted data, it was necessary to smooth these ripples.

Simple moving-average smoothing was applied to the measured data. A new value was assigned to each voxel, which was the mean of the values corresponding to five voxels in the original data grid along the smooth processing axis: the current voxel, two preceding voxels, and two subsequent voxels. The span is always set to an odd number in moving-average smoothing, and in this case, it was five voxels. This smoothing process was applied to the measured data along the x, y, and z axes and covered the entire data grid.

The measured results had a different scale than the PET-RV prediction results. Our PET results were acquired by performing offline PET, the PET/CT device was designed for nuclear medicine data acquisition and image reconstruction. Thus, the PET activity after irradiation of the phantom was much lower than that of a patient injected with positron-emitting medicine. Due to the low counting rate and noise effect, our measured PET data could not provide real PET activity values in the phantom. Furthermore, since the prediction script required some time-related parameters to calibrate the calculation results with the irradiation time course, this process could not be controlled with complete accuracy, so we just considered the shapes of the distributions rather than the actual PET values.

Before performing the comparison, it was necessary to normalize the measured data to the prediction results. A factor was multiplied to the measured data to rescale the value so that measured results and the prediction results will have similar amplitude, and this factor was determined based on the ratio between the two sets of results. To find this ratio, it was necessary to compare the PET values of both sets in a flat region, where flat is defined as the activity distribution gradient being below a certain threshold. First, the data points with PET values >50% of the maximum were selected, since in the data grid, many points had values of 0, forming a pseudo-flat region, but would not have been appropriate to include in the comparison. Then, to filter the points in the flat region, the gradients of all the points were calculated by finding their slopes in the x, y, and z directions, and for each point, if the three slopes were all less than a predefined threshold, then the point was identified as a flat point. This filtering process was conducted to generate a position list containing all the points in the PET-RV prediction results that were defined as flat.

Then, the filtering process was applied to the measurements. Since the predicted and measured results had the same data structure, it was not necessary to search all the points in the measured PET data grid. Instead, only the positions recorded in the list from PET-RV filtering were searched, and it was checked whether the gradient at each point was also less than the threshold in the measured data grid. Then, a new position list was generated to record the points that were designated as flat in both the measurements and PET-RV prediction results.

After identifying the common flat region, the multiplicative factor could be generated by calculating the ratio between the measured and predicted values for each point, compiling a ratio list, and finding the mean value of this ratio list. The mean ratio of this list was then applied to the measured data.

### Depth comparison of PET-RV predictions and measurements

**Figure 2:**
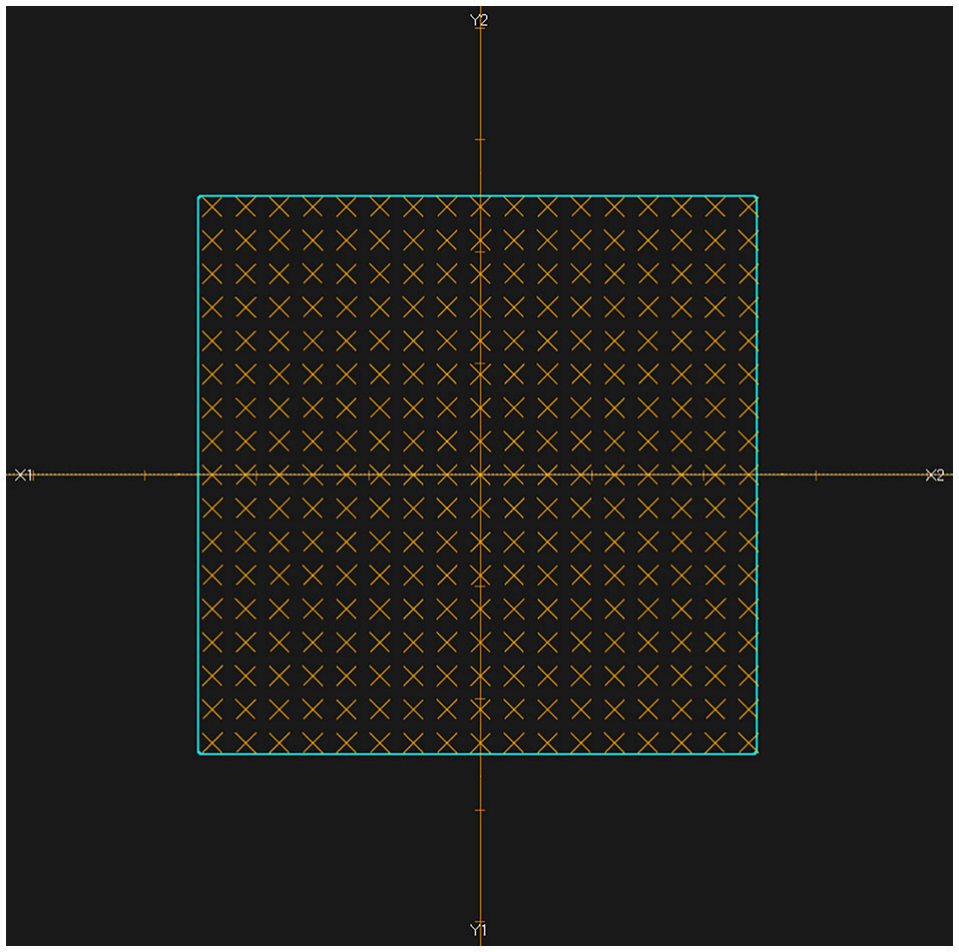
BEV of irradiated ROI (cyan). All of the scoring lines are perpendicular to the transverse section and limited inside the cyan area. The dimensions of the cyan area are 5 cm × 5 cm, and the scoring lines were chosen to have an interval of 3 mm.

The activity distribution depth scoring line is set parallel to the beam line. In this experiment, the plans were designed as doses delivered uniformly to a cubic region of interest (ROI), so in the transverse beam eye view (BEV), we set the scoring line uniformly inside the transverse section of the ROI. Each CTV had a 5 cm × 5 cm transverse section perpendicular to the beam line, which was then set as the scoring section. As the data grid had voxel bins with dimensions of 3 mm × 3 mm × 3 mm, the interval along the scoring line was also chosen to be 3 mm, to avoid performing unnecessary interpolation calculations, as shown in Figure 2.

For each scoring line inside the scoring section, two position-PET curves were drawn, one each for the predicted and measured PET data. Then, the depth difference on each line could be evaluated and the statistical information about the global depth error in each case could be obtained.

**Figure 3:**
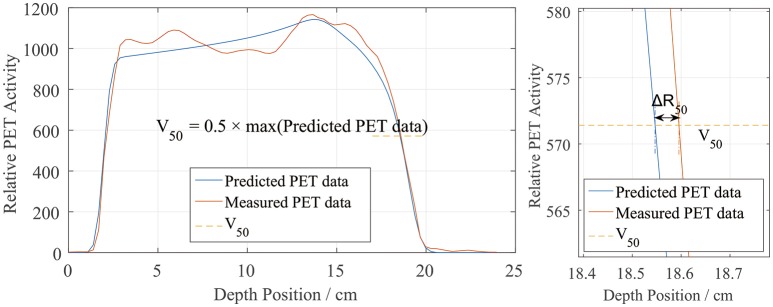
Sample depth score curve (**left**) and the definition of ΔR_50_ on the scoring sample (**right**). V_50_ is defined by the predicted PET data.

The most commonly used method of evaluating the range error between two PET curves is to locate the position of the half-maximum point R_50_ on the scoring curve at the distal fall-off edge (2, 7, 10, 12, 16, 18). This method was also applied in this study, as shown in Figure 3. First, the global maximum value of the PET-RV prediction data was obtained, and half of this maximum value was calculated and recorded as V_50_. Since the measured results were already normalized to the predicted results, the entire position locating process was based on this V_50_ calculated from the predicted data. Then, for each position-PET curve, the position on the curve with a corresponding PET value equal to V_50_ was identified. Since both the entrance and distal fall-off edges have positions with values equal to V_50_ in a PET curve, it was also necessary to check that each point was obtained when the PET value was decreasing rather than increasing. After doing so, the point on the distal fall-off edge with a PET value equal to V_50_ was defined as R_50_ of the curve. Finally, after acquiring R_50_ for both the predicted and measured PET curves, we calculated ΔR_50_ for each scoring line as the depth error between the two PET curves along the scoring line:

$$\begin{array}{l} \Delta R_{50,depth}=R_{50,pred}-R_{50,meas} \end{array}$$

After calculating all of the depth errors, we obtained the mean depth error in each irradiation case.

### Lateral comparison of PET-RV predictions and measurements

**Figure 4:**
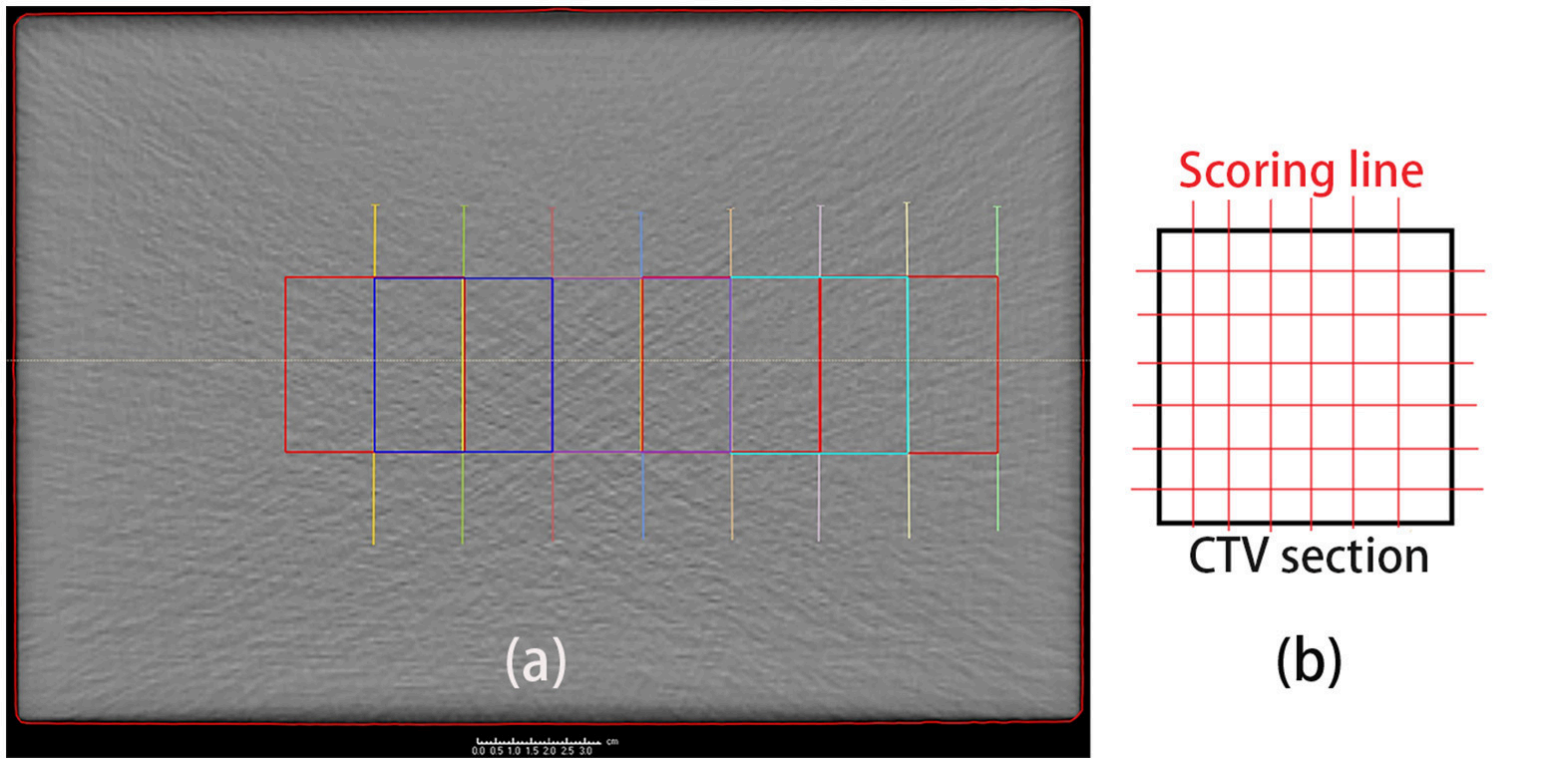
**(a)** Set scoring layer positions. The depth ranges from 2.5 cm (green) to 20 cm (yellow), in 2.5 cm steps, from right (entrance) to left. **(b)** Scoring lines in each CTV section. To obtain the entire lateral curves, the scoring lines were extended to outside the CTV section.

Besides the depth, the lateral PET profiles needed to be checked as well. Lateral profile scoring layers were set at different depths from the beam entrance. As the dose and activity depth varied among the different cases, the profile scoring layers were set at depths ranging from 2.5 to 20 cm in 2.5 cm steps starting from the entrance, with eight layers in total (Figure 4a). Since the experiment cases have different dose depth, for example, in the case of 10 cm dose depth, scoring layers at depth deeper than 10 cm cannot score valid data, thus only 4 layers with depth 2.5, 5.0, 7.5, and 10 cm will be employed for the lateral scoring in this case. Different scoring layers will be employed in different cases.

The lateral scoring section was a square with two basis vectors perpendicular to the beam direction vector. The section size was 5 cm, and lateral scoring lines were set parallel to the basis vectors, in intervals of 3 mm, which was the same as the data voxel size (Figure 4b). Along each scoring line, we obtained the lateral distribution curves for the PET predictions and measurements.

**Figure 5:**
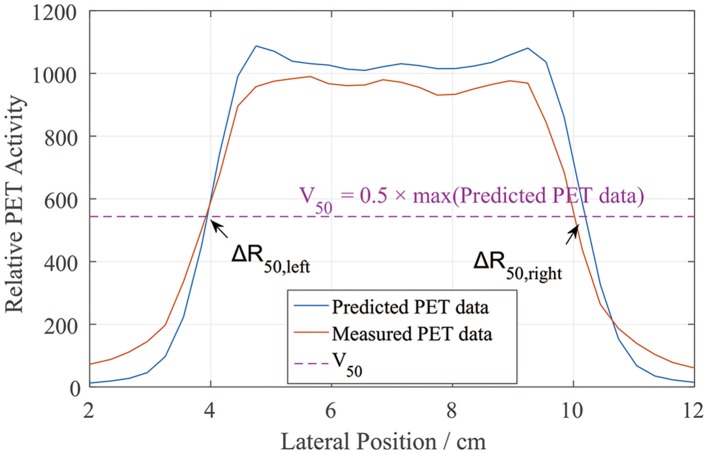
PET lateral scoring curves, where the lateral position errors between the PET-RV predictions and measurements were found on the left and right sides.

We similarly defined the positions of the left and right sides of the curves by locating R_50_ (2). Unlike in the depth error analysis, R_50_ could be located on either side of the lateral PET curves (Figure 5). Similarly, we found half of the maximum value on the predicted data curve and defined it as V_50_. Then, on each lateral curve, we identified the positions of R_50_ on both the left and right sides. To calculate the lateral error along each scoring line, ΔR_50_ was calculated using

$$\begin{array}{l} \Delta R_{50,lateral}=|R_{50,pred}-R_{50,meas}| \end{array}$$

This equation was applied to both the left and right sides. The absolute value was taken to verify the relative position errors rather than just compare the distribution widths. After collecting all of the ΔR_50_ values, we calculated the mean lateral position error and deviation for each scoring layer.

### Gamma index analysis

Gamma index analysis is also applied to the data comparison. Similar to the method applied in dose verification (19), we can also compare the data of prediction and measurement point by point through the entire data grid. Gamma analysis method for dose comparison evaluate both dose difference and distance-to-agreement (DTA), and calculate a γ value for each data point of the prediction data. If a point is γ <1, then this point is acceptable with given criteria, otherwise, the prediction and measurement distribution data at this point is not agreed. Detail of the γ calculation can refer to other articles (19, 20) and will not be mentioned here. Our point value difference and DTA tolerance are set to 3% and 3 mm, and the analyzing data grid has size of 36.90 cm × 23.10 cm × 21.60 cm with voxel size 3 mm × 3 mm × 3 mm. All the radiation plans have a gamma index analysis, the prediction activity distributions are verified to the corresponding measurement data, and the gamma passing rates of each plan are calculated.

## Results

### Global view of plan design and PET normalization

**Figure 6:**
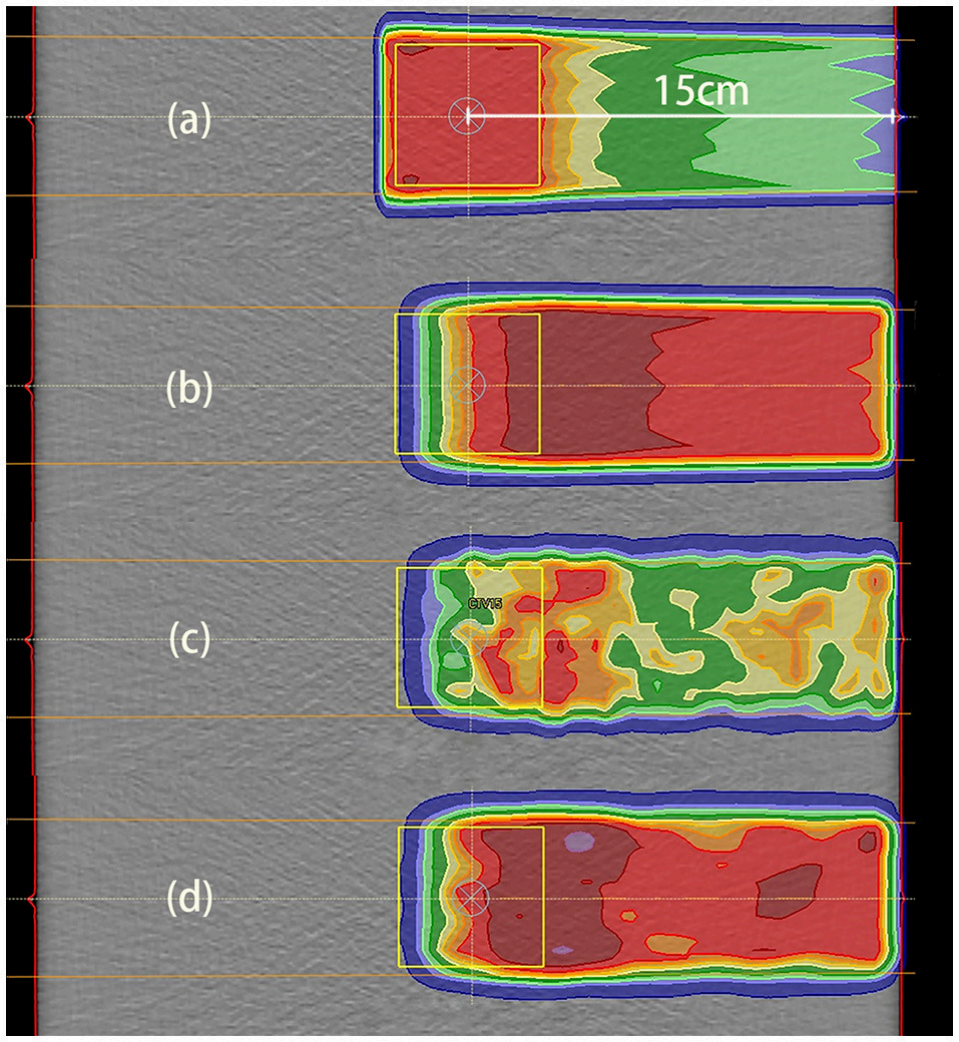
Data distributions in the 2 Gy case with a depth of 15 cm. **(a)** Dose distribution, where the dose was designed for uniform distribution throughout the CTV; **(b)** PET results predicted using PET-RV; **(c)** original measured PET data; **(d)** measured PET data after smoothing and normalization. **(b,d)** have similar ranges and values, which means that the measured data agreed better with the predicted data after smoothing and normalization.

Our irradiation plans were designed for uniform dose delivery to a cubic CTV. The dose distribution for the 2 Gy case with a depth of 15 cm is depicted in Figure 6a.

The CTV for dose distribution was designed as a cube with dimensions of 5 cm × 5 cm × 5 cm, and the depth of the CTV center below the entrance was 15 cm in this case. Thus, the distal dose fall-off edge depth was actually near 17.5 cm, 2.5 cm deeper than the CTV center.

The distal fall-off edge of the activity distribution had a depth less than that of the dose distribution due to the channel cut-off. For each phantom case, the calculations performed to make the prediction took <30 s. As shown in Figures 6b–d, the predicted PET data have a distribution range similar to that of the measured PET data but differ in absolute value, and after smoothing and normalization, both the ranges and PET values are similar.

**Figure 7:**
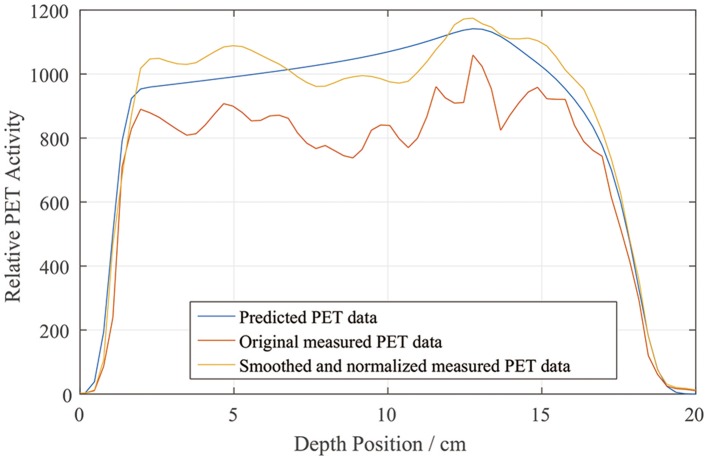
Sample depth scoring curves for predicted, measured, and smoothed and normalized PET data.

We drew a sample scoring line to illustrate how the smoothing and normalization processes changed the measured PET curves. In Figure 7, it is evident that the measured curve after smoothing and normalization matches the predicted curve more closely, especially on the distal fall-off edge.

### PET-RV prediction and measurement depth comparison

**Figure 8:**
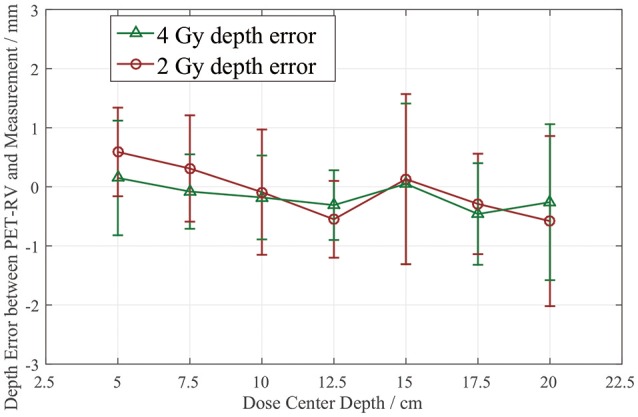
PET activity depth error Δ*R*_50_ for 2 Gy and 4 Gy cases with different dose center depths.

The depth errors between the PET-RV predictions and measurements in the 2 and 4 Gy cases are presented in Table 1 and Figure 8.

**Table 1:** Depth error (PMMA) for different cases.

**Depth/cm**	**5**	**7.5**	**10**	**12.5**	**15**	**17.5**	**20**
**2 Gy**							
Δ*R*_50_/mm	0.59 ± 0.75	0.31 ± 0.90	−0.09 ± 1.06	−0.55 ± 0.65	0.13 ± 1.44	−0.29 ± 0.85	−0.58 ± 1.44
**4 Gy**							
Δ*R*_50_/mm	0.15 ± 0.97	−0.08 ± 0.63	−0.18 ± 0.71	−0.31 ± 0.59	0.05 ± 1.36	−0.46 ± 0.86	−0.26 ± 1.32

From Figure 8, it can be seen that the depth error Δ*R*_50_ between the PET-RV predictions and measurements has a mean value between 1 and −1 mm in the PMMA phantom in each case, considering that with the error bars, the absolute value of Δ*R*_50_ is <2 mm in most cases. The 2 and 4 Gy dose cases have similar mean depth errors, but with a higher dose, more protons are delivered, causing a high-intensity PET signal with a better signal-to-noise ratio to be generated in the 4 Gy cases. Consequently, the error bars in the 4 Gy cases are smaller than those in the 2 Gy cases. This finding is relatively acceptable compared with those obtained in other research (21, 22) in which patient data were employed, because a phantom is a uniform and static material and will yield better results than the human body.

### Lateral comparison of PET-RV predictions and measurements

**Figure 9:**
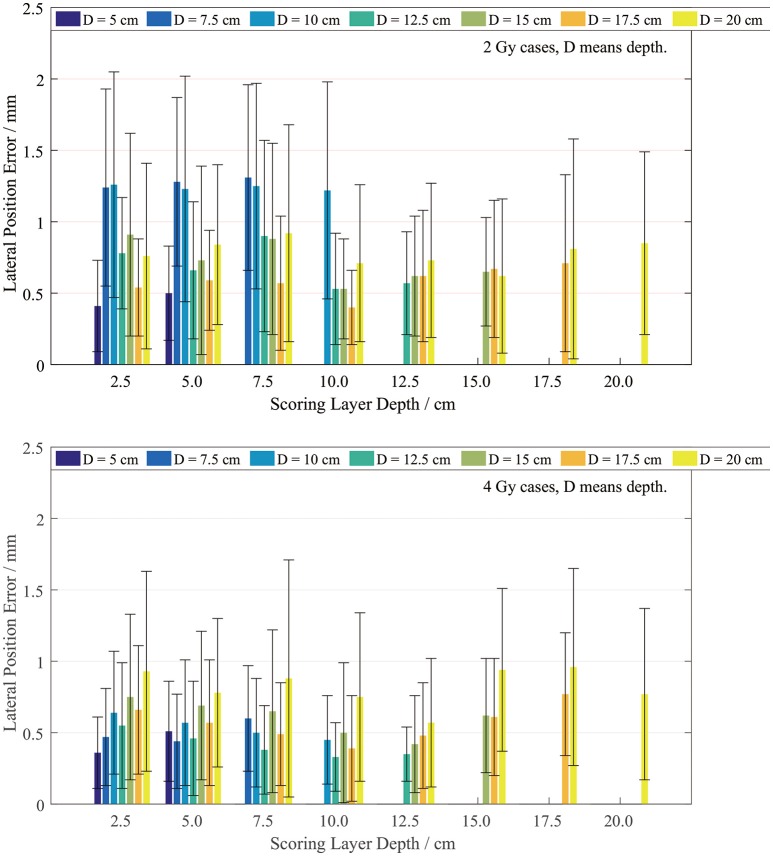
Lateral errors for the 2 Gy **(top)** and 4 Gy **(bottom)** cases with different depths.

The lateral width error was scored on layers with different depths. Since our plan series had different dose depths, the lateral scoring layers were set only at the positions with valid PET data. We scored the position errors between the PET-RV predictions and measurements, and the results are presented in Figure 9.

It is evident that the mean lateral error is <1 mm in most cases, especially the 4 Gy cases. The lateral position error in the 4 Gy cases is smaller than that in the 2 Gy cases since, as mentioned in section PET-RV Prediction and Measurement Depth Comparison, a higher irradiation dose introduces lower uncertainty.

### Gamma index analysis result

The gamma passing rates of each case are shown in Table 2. All the cases have a passing rate over 95%, this rate meets the clinical requirement of dose verification, while in PET data gamma analysis the rate requirement maybe different to the dose requirement. With the dose depth increasing, the passing rates have a overall decreasing trend because the increasing depth will create more voxels with non-zero activity values, with the effect of noise signal, the passing rate is sure to reduce. Under the same depth condition, the 4 Gy cases always have a higher passing rate than 2 Gy cases, because the higher delivery dose will reduce the effect of noise.

**Table 2:** The gamma passing rate of each radiation plan.

**Depth/cm**	**5**	**7.5**	**10**	**12.5**	**15**	**17.5**	**20**
**2 Gy**							
Passing Rate/%	99.43	98.47	98.53	97.38	97.74	96.21	95.86
**4 Gy**							
Passing Rate/%	99.54	99.26	99.30	97.98	98.59	96.70	95.94

## Discussion

### Normalization method and threshold

As mentioned in section Measured Data Smoothing and Normalization, neither the predicted nor measured PET data present the actual PET activity values, and normalization is necessary before comparison. Besides the flat region normalization process introduced in section Measured Data Smoothing and Normalization, there is another normalization method that can be employed to obtain the ratio. In this method, the sums of the predicted and measured data in the entire data grid are calculated, and the ratio of these two sums is set as the normalizing factor. This process is much simpler than flat region normalization, but the errors of the depth comparison results with sum normalization are larger than those with flat region normalization. Typically, the mean depth error is >1 mm when sum normalization is applied. The reason for this difference is that the sum of the data over the entire grid includes all of the voxels in the data grid, which will introduce the effects of noise in blank areas, while the flat region is filtered and selected in the irradiation area, so the factor calculated by flat region normalization yields better depth comparison results.

In flat region normalization, the gradient threshold for the “flat” definition should be chosen with assessment. In the normalization process described in section Measured Data Smoothing and Normalization, we first identified voxels with PET values greater than half of the maximum and recorded the total number of these voxels. We then searched both the predicted and measured data grids and obtained the points in the flat region. The number of flat points reflected how the threshold worked, since with a different gradient threshold, the size of the flat region would differ as well. The threshold was defined as how much the PET value changed within 1 mm, and the unit was 1% of the maximum PET value per millimeter, which was recorded as TH1:

$$\begin{array}{l} \text{TH}1=\frac{1\%\times PET_{\text{max}}}{1mm} \end{array}$$

Thus, we determined the threshold using THx = factor × TH1 and tried different factor values in the 2 Gy dose, 15 cm dose depth and 4 Gy dose, 15 cm dose depth cases, which yielded the results presented in Table 3.

**Table 3:** Number of flat points and ratio (number/total) for all of the voxels.

**Trial case**	**Total**	**Factor**									
		**0.7**	**0.8**	**0.9**	**1.0**	**1.1**	**1.2**	**1.3**	**1.4**	**1.5**	**1.6**
2 Gy dose, 15 cm dose depth	21,977	5,322 (0.242)	6,503 (0.296)	7,387 (0.336)	8,083 (0.368)	8,633 (0.393)	9,092 (0.414)	9,407 (0.428)	9,649 (0.439)	9,982 (0.454)	10,252 (0.466)
4 Gy dose, 15 cm dose depth	22,410	6,049 (0.270)	7,006 (0.313)	7,820 (0.349)	8,331 (0.372)	8,848 (0.395)	9,349 (0.417)	9,741 (0.435)	10,082 (0.450)	10,496 (0.468)	10,815 (0.483)

As the factor increases, the threshold increases, as does the applicable voxel number. The data in Table 3 demonstrate that when the factor is 1.4, the number of flat voxels has a lower rate of increase than it does with the other factor values. In addition, when the factor was >2.0 or <0.5, the comparison did not exhibit good fitness, so we set the normalizing gradient threshold factor to 1.4 and applied the corresponding gradient threshold in each case in the normalization process.

### Definitions of PET range and position

**Figure 10:**
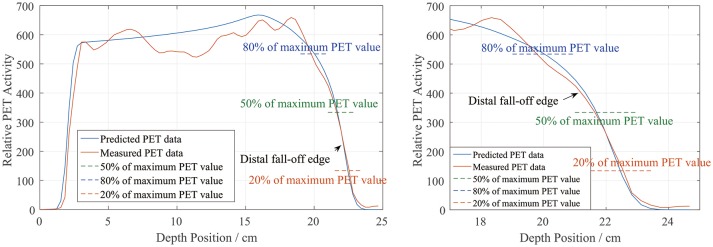
PET activity depth scoring sample along the beam direction (**left**) and the curve at the distal fall-off edge (**right**). The predicted and measured data show good fitness at the distal fall-off edge after smoothing and normalization.

In the depth and lateral verifications, the PET distal position was defined as 50% of the maximum PET value on the distal falling edge. In our data preprocessing, we attempted to define the PET activity distribution ranges using the positions corresponding to 80, 50, and 20% of the maximum, which were recorded as R_80_, R_50_, and R_20_, respectively. Figure 10 shows a sample activity depth scoring curve. On the distal edge, R_80_ would be easily affected by the ripples in the measured curve, even though the smoothing process was already applied to the measured data. R_20_ seems acceptable in the presented case, but it could also be affected by the noise in the measured data (12). Since R_20_ was near the base line, the noise on the base line would have affected the edge after normalization, so we did not use R_20_ for verification.

There is also a range verification method that involves shifting the curve to achieve good fitness between the predicted and measured curves (12). However, in our phantom cases, the measured data were normalized before performing the comparison and information about the actual PET values may have been lost. Even if we had forced a shift, the measured data would have exhibited numerous ripples that would have affected the comparison substantially and covered the useful information on the distal edge. Therefore, we finally choose R_50_ to define the PET range for both depth and lateral verification.

### Time course for *in vivo* cases

For now, our experiment is executed on a homogenous solid phantom with only one field, which means we do not need to consider for the washout yet, and the spots scanning time course is also simplified with fixed particle delivery rate, spot motion speed and layer shift time. This process shows a reasonable result in this phantom because of the small radiation field and uniform dose distribution. While in patient application, radiation field in some treatment cases will be large and the spending time for one field is even more than 15 min. Under this condition, different part of the generated positron isotope will not share the same cooling time. One possible method to solve this problem is get the spots delivery record from the accelerator, the calculation process can apply different decay time for each spot generated positron isotope then. Another method, if the spot record is hard or not possible to access, then we need to construct different spot scanning time course model for different kind of treatment cases.

Washout parameter is another important factor for the prediction if the calculation is applied *in vivo*. Mizuno (23) provides a mathematical formulation for *in vivo* washout process and Parodi (16, 24) provides parameters for the formulation. In this phantom experiment the washout process is not needed, only physical decay is applied in prediction calculation. For *in vivo* applications, activity washout can be calculated with different time parameters depend on the HU value of the position. This work needs further investigation.

## Conclusion

In this study, we developed a process for comparing predicted and measured PET images and verified the correctness of a PET prediction module. It was necessary to smooth and normalize the measured PET data for better fitness with the predicted data before comparison. The depth comparison between the PET-RV predictions and measurements yielded |Δ*R*_50_| of no more than 2 mm with the error bars considered. The lateral position error was also no more than 2 mm. This comparison and verification process was effective and demonstrated that the predictions could fit the PET measurement range within an uncertainty of 2 mm in PMMA.

For the prediction process, the PBS time course was also a parameter. Since irradiation history record analysis was not implemented in the script, we used a fixed delivery time model for the particle delivery. These fixed time parameters may not have fit the actual plan execution processes perfectly, so the predicted data distributions differed slightly from the measured data. However, we performed normalization before comparing the two types of data, so this difference did not affect the final comparison. Another problem related to the time course is the washout parameter. We employed a solid phantom, so this issue was not problematic, but it will need to be dealt with for further *in vivo* research.

Verification using a human-like model and patient body will be performed in future investigations. This PET verification can indicate whether the treatment plan is executed as expected by comparing measured and predicted PET data. Thus, it allows doctors to ascertain whether the delivery dose depth is too deep and whether the organ at risk (OAR) is covered by the dose distribution. This verification is applied on the first fraction of treatment and physicians can tune the treatment plan before delivering the remaining fractions.

## Author contributions

JyZ: manuscript production, verification process designing, and irradiation experiment execution; YL: irradiation plan designing and experiment assistance; JgZ and LS: PET image acquisition; YS: experiment beam time arrangement; WH and WW: depth range verification method suggestion; RZ: manuscript revision; JC and JL: financial support.

### Conflict of interest statement

The authors declare that the research was conducted in the absence of any commercial or financial relationships that could be construed as a potential conflict of interest.

## References

[1] Brada M Pijls-Johannesma M De Ruysscher D. Proton therapy in clinical practice: current clinical evidence. J Clin Oncol. (2007) 25:965–70. 10.1200/JCO.2006.10.0131 17350945

[2] Parodi K Harald P Cascio E Flanz JB Bonab AA Alpert NM. PET/CT imaging for treatment verification after proton therapy: a study with plastic phantoms and metallic implants. Med Phys. (2007) 34:419–35. 10.1118/1.2401042 17388158 PMC2292642

[3] Enghardt W Crespo P Fiedler F Hinz R Parodi K Pawelke J Charged hadron tumour therapy monitoring by means of PET. Nuclear Inst Methods Phys Res Sect A (2004) 525:284–8. 10.1016/j.nima.2004.03.128

[4] Zhu XP El FG. Proton therapy verification with PET imaging. Theranostics (2013) 3:731–40. 10.7150/thno.5162 24312147 PMC3840408

[5] Barschall HH Chadwick MB Jones DTL Meulders JP Schuhmacher H Young PG Appendix D: proton data tables. J Int Commission Radiat Units Meas. (2000) os32:166–236. 10.1093/jicru/os32.2.166

[6] Beebe-wang JJ Dilmanian FA Peggs SG Schlyer DJ Vaska P Feasibility of positron emission tomography of dose distribution in proton-beam cancer therapy. In: 8th European Particle Accelerator Conference, Paris (2002).

[7] Knopf A Parodi K Bortfeld T Shih HA Paganetti H Systematic analysis of biological and physical limitations of proton beam range verification with offline PET/CT scans. Phys Med Biol. (2009) 4:4477–95. 10.1088/0031-9155/54/14/008 19556685

[8] Bauer J Unholtz D Kurz C Parodi K. An experimental approach to improve the Monte Carlo modelling of offline PET/CT-imaging of positron emitters induced by scanned proton beams. Phys Med Biol. (2013) 58:5193–13. 10.1088/0031-9155/58/15/5193 23835872

[9] Frey K Bauer J Unholtz D Kurz C Krämer M Bortfeld T. TPSPET-A TPS-based approach for *in vivo* dose verification with PET in proton therapy. Phys Med Biol. (2014) 59:1–21. 10.1088/0031-9155/59/1/1 24323977

[10] Parodi K Ponisch F Enghardt W Experimental study on the feasibility of in-beam PET for accurate monitoring of proton therapy. IEEE Trans Nucl Sci. (2005) 52:778–86. 10.1109/TNS.2005.850950

[11] Parodi K Enghardt W Haberer T. In-beam PET measurements of beta(+) radioactivity induced by proton beams. Phys Med Biol. (2002) 47:21–36. 10.1088/0031-9155/47/1/302 11814225

[12] Knopf A Parodi K Paganetti H Cascio E Bonab A Bortfeld T. Quantitative assessment of the physical potential of proton beam range verification with PET/CT. Phys Med Biol. (2008) 53:4137–51. 10.1088/0031-9155/53/15/009 18635897

[13] Combs SE Jaekel O Haberer T Debus J. Particle therapy at the Heidelberg Ion Therapy Center (HIT) - integrated research-driven university-hospital-based radiation oncology service in Heidelberg, Germany. Radiother Oncol. (2010) 95:41–44. 10.1016/j.radonc.2010.02.016 20227124

[14] Jakoby BW Bercier Y Conti M Casey ME Bendriem B Townsend DW. Physical and clinical performance of the mCT time-of-flight PET/CT scanner. Phys Med Biol. (2011) 56:2375–89. 10.1088/0031-9155/56/8/004 21427485

[15] Chen W Bauer J Kurz C Tessonnier T Handrack J Haberer T A dedicated software application for treatment verification with off-line PET/CT imaging at the Heidelberg Ion Beam Therapy Center. J Phys Conf Ser. (2017) 777:12–21. 10.1088/1742-6596/777/1/012021

[16] Parodi K Harald P Shih HA Michaud S Loeffler JS Delaney TF. Patient study of *in vivo* verification of beam delivery and range, using positron emission tomography and computed tomography imaging after proton therapy. Int J Radiat Oncol Biol Phys. (2007) 68:920–34. 10.1016/j.ijrobp.2007.01.063 17544003 PMC2047826

[17] Hudson HM Larkin RS. Accelerated image reconstruction using ordered subsets of projection data. IEEE Trans Med Imaging (1994) 13:601–9. 10.1109/42.363108 18218538

[18] Nishio T Sato T Kitamura H Murakami K Ogino T. Distributions of β+ decayed nuclei generated in the CH2 and H2O targets by the target nuclear fragment reaction using therapeutic MONO and SOBP proton beam. Med Phys. (2005) 32:1070–82. 10.1118/1.1879692 15895592

[19] Low DA Harms WB Mutic S Purdy JA. A technique for the quantitative evaluation of dose distributions. Med Phys. (1998) 25:656–61. 10.1118/1.598248 9608475

[20] Depuydt T Van EA Huyskens DP. A quantitative evaluation of IMRT dose distributions: refinement and clinical assessment of the gamma evaluation. Radiother Oncol. (2002) 62:309–19. 10.1016/S0167-8140(01)00497-2 12175562

[21] Min CH Zhu X Grogg K El Fakhri G Winey B Paganetti H. A recommendation on how to analyze in-room PET for *in vivo* proton range verification using a distal PET surface method. Technol Cancer Res Treat. (2014) 14:320–5. 10.1177/1533034614547457 25246517 PMC4898041

[22] Wen CH Indelicato DJ Vargas C Duvvuri S Li Z Palta J *In vivo* verification of proton beam path by using post-treatment PET/CT imaging. Med Phys. (2009) 36:4136–46. 10.1118/1.3193677 19810487

[23] Mizuno H Tomitani T Kanazawa M Kitagawa A Pawelke J Iseki Y. Washout measurement of radioisotope implanted by radioactive beams in the rabbit. Phys Med Biol. (2003) 48:2269–81. 10.1088/0031-9155/48/15/302 12953897

[24] Parodi K Bortfeld T Haberer T. Comparison between in-beam and offline positron emission tomography imaging of proton and carbon ion therapeutic irradiation at synchrotron- and cyclotron-based facilities. Int J Radiat Oncol Biol Phys. (2008) 71:945–56. 10.1016/j.ijrobp.2008.02.033 18514787

